# Prevalence of early and late onset of chronic diseases and multimorbidity and its association with physical, mental and functional health among older Indian adults

**DOI:** 10.1186/s12877-023-04264-8

**Published:** 2023-09-14

**Authors:** Waquar Ahmed, T. Muhammad, K. Muneera

**Affiliations:** 1 School of Health Systems Studies, Tata Institute of Social Sciences, Mumbai, India; 2 Department of Family & Generations, International Institute for Population Sciences, Mumbai, India; 3 National Institute of Technology, Calicut, Kerala 673601 India

**Keywords:** Age at onset of disease, Physical health, Mental health, Functional health, Older adults

## Abstract

**Background:**

Identifying people with early and late onset of chronic conditions might help target the subpopulations that are more vulnerable to negative mental, physical and functional health outcomes. The current study aimed to examine the association of early and late onset of chronic single and multiple morbidities with self-perceived physical and mental health, functional limitations and physical inactivity among older Indian adults.

**Methods:**

Cross-sectional study was conducted using data from the Longitudinal Ageing Study in India (LASI) Wave 1 (2017–2018). The total sample size for the present study was 31,386 older adults age 60 years or older. Multivariable binary logistic regression analysis was used to establish the association between the outcomes (poor perceived physical/mental health, functional difficulty and physical inactivity) and explanatory variables (early [ = < 50 years of age] and late [> 50 years]) onset of chronic illnesses such as hypertension, diabetes, heart attack, heart disease, stroke, cancer, lung disease, arthritis, osteoporosis and psychiatric disease).

**Results:**

Overall, 24.21% of the sample population had poor self-perceived physical health, whereas 8.67% of participants had poor self-perceived mental health. The prevalence of difficulty in ADL, difficulty in IADL, and physical inactivity was 23.77%, 48.36%, and 68.9%, respectively. Odds of poor perceived mental health were higher for the respondents with early as well as late onset of hypertension, stroke, and arthritis; while individuals with late onset of diabetes, and heart disease had higher odds of poor perceived mental health than those without chronic disease. Individuals with early onset of single morbidity were more likely to report ADL difficulty (adjusted odds ratio [AOR]: 1.33, confidence interval [CI]: 1.06–1.67); while those with late onset of single (AOR: 1.34, CI: 1.17–1.53) and multimorbidity (AOR: 1.91, CI: 1.63–2.24) were more likely to report ADL difficulty compared with individuals without morbidity. Individuals with early as well as late-onset of multimorbidity had more than two times higher odds of reporting poor physical health, poor mental health and IADL difficulty compared with individuals without chronic disease.

**Conclusions:**

The present study revealed that early and/or late onset of chronic single and/or multiple morbidities significantly predicted poor self-perceived physical and mental health, functional limitations and physical inactivity among older Indian adults. The findings further suggest that late onset of chronic diseases such as cancer and stroke and multi-morbidity had stronger associations with physical inactivity that may help identify high risk groups for screening and support.

## Background

Chronic diseases represent the leading cause of mortality worldwide, and most deaths from chronic diseases occur in middle-to-low-income nations [1]. Due to changes in lifestyle and dietary habits, the prevalence of chronic conditions has become a major public health concern across the globe. In India, individuals aged 45 years and above accounted for more than half of the burden of non-communicable diseases (NCDs) [2]. Chronic conditions are associated with poor lifestyles in old age [3, 4]. Additionally, it has been shown to have negative consequences on functional, psychological, and cognitive health, mortality rates, and healthcare spending. [5–8].

Importantly, chronic diseases have been shown to be associated with the self-perceived physical health of older individuals. Existing literature suggests that having a single long-term disease or the number of diseases has a negative impact on older people’s rating of physical health [9–11]. The studies, however, highlight that multiple diseases can have a greater impact on the perceived health of individuals because of subjective representation and consequences of multiple illnesses, unlike single disease [12–14]. There is also ample evidence of the relationship between chronic diseases and mental illnesses. The number of chronic diseases modify the physiology of the brain and lead to mental illness, just as having a mental illness increases the likelihood of having comorbid diseases [15]. On the other hand, interactions between age and chronic diseases show that people who have chronic illnesses early in life are likely to have more depressive symptoms than people who get illnesses later in life. Likewise, disability increases depression symptoms when it occurs earlier in life [16]. According to one comparative study of symptoms and risk factors in the early and late onset of depression, vascular diseases were found to be associated with the late onset of depression [17].

The risk of psychological distress is higher among long-term cancer survivors. Additionally, people with comorbid illnesses and those who had trouble doing any IADLs were far more likely to have serious psychological distress (SPD). Compared to responders who had never been diagnosed with cancer, long-term cancer survivors had a significantly greater prevalence of SPD [18]. Although the majority of long-term cancer survivors successfully adjust to life after cancer and may even have positive psychological effects from dealing with their cancer [19–21]. Cancer diagnosis and treatment outcomes can have an impact on a person’s physical and psychological well-being. In addition to delayed physical effects that manifest many years after the end of therapy, cancer treatment can also result in immediate physical deficiencies [22–24]. Similarly, in comparison to people without respiratory disease, those with respiratory disease appear to have a higher prevalence of comorbid depression [25].

In a previous study, it was reported that increased chances of functional impairment were more associated with cancer, diabetes, and incontinence in males aged 70–79 years, whereas pulmonary illness and diabetes were found to be strongly associated in women [26]. Chronic multimorbidity decreases older persons’ likelihood of having better physical functioning [27]. Arthritis, diabetes, cardiovascular disease, osteoporosis, lung diseases, and high blood pressure are the most prevalent illnesses associated with functional limitation [28–30]. According to Klijis et al., cardiovascular and musculoskeletal diseases were associated with impairment in activities of daily living and instrumental activities of daily living among older people [29]. Similarly, among Brazilian older adults, stroke, hypertension, diabetes, and arthritis were the most prevalent chronic condition for disability [28]. Finally, older people with chronic conditions such as diabetes, high blood pressure, heart disease, stroke and cancer and other mental illnesses were more likely to report physical inactivity [31], and studies suggest that targeting people with chronic diseases will reduce the impact of physical inactivity on other health outcomes [32, 33].

Therefore, identifying people with early and late onset of chronic conditions might help target the subpopulations that are more vulnerable to negative mental, physical and functional health outcomes. Especially, early/late onset of chronic diseases and associated adverse health outcomes necessitates effective treatment approaches. Understanding the linkage between the age of onset of diseases and health outcomes is crucial to address patient concerns, develop personalized treatment strategies and support tailored to patients with early and late onset of chronic disease and multimorbidity, and implement evidence-based interventions to optimize outcomes not only in terms of disease treatment but also considering quality of life, physical, mental and functional outcomes. Thus, the current study aimed to examine the association of early and late onset of chronic single and multiple morbidities with self-perceived physical and mental health, functional limitations and physical inactivity among older Indian adults.

## Methods

### Data

Data from the survey of the Longitudinal Ageing Study in India (LASI) Wave 1 (2017–2018) were considered for the current analysis. The survey collected information on the health, economic, social, and demographic aspects of India’s ageing population as well as its consequences. The LASI is a nationally representative survey that included 72,250 individuals who were 45 years of age or older along with their spouses (irrespective of age) in all Indian states and union territories of India except Sikkim. The LASI employs a multistage stratified area probability cluster sampling to select the eventual units of observation. The LASI provides information on chronic health conditions, biomarkers, symptom-based health conditions, and functional and mental health. The LASI survey was carried out using a three-stage sampling design and a four-stage sampling design in rural areas and urban areas, respectively. In each state/UT, Primary Sampling Units (PSUs) were chosen in the first stage, while villages in rural areas and wards in urban areas were chosen in the selected PSUs in the second stage. Households were selected from each identified village in the third stage; however, sampling in urban areas required an additional stage, which comprised the randomly selecting one Census Enumeration Block (CEB) in each urban area. From each CEB, households were selected in the fourth stage. The main goal was to select a representative sample at each stage of sample selection.

**Fig. 1:**
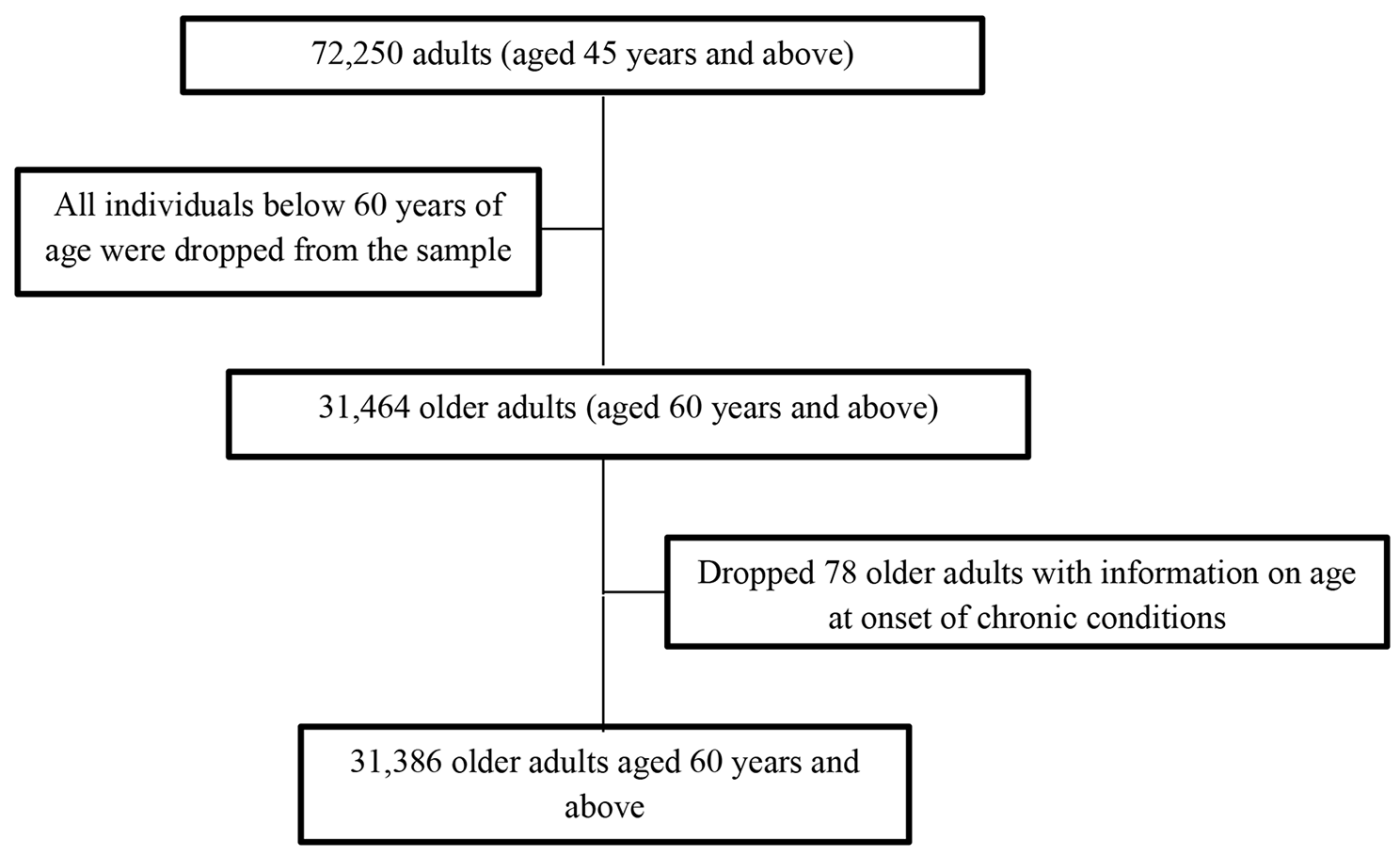
Sample selection criteria for this study

The LASI used computer-assisted personal interview (CAPI) technology for the data collection. This method required field teams to be outfitted with laptop computers pre-loaded with survey questions asked of respondents in a face-to-face interview. The report contains considerable information on the survey’s design, data collection, and methodology. The data are available at The Gateway to Global Aging Data (https://g2aging.org/ ). The present study is based on eligible respondents who are aged 60 years and above. The total sample size for the present study after dropping the missing cases (n = 78) was 31,386 older adults aged 60 years or older (Fig. 1**)**.

## Measures

### Outcome variables

#### Poor perceived physical health

Poor self-rated health (SRH) was available on a scale of five, which represents good (very good, good, fair) and poor (poor and very poor). Further, poor SRH was coded as yes and no.

#### Poor perceived mental health

Poor perceived mental health was assessed using the Short Form Composite International Diagnostic Interview (CIDI-SF) with a cut-off score of 3 or more on the scale of 0–10. This scale is widely used in population-based health surveys [34]. The scale is a fully-structured diagnostic interview based on the Diagnostic and Statistical Manual of Mental Disorders (DSM) criteria for major depressive episode and validated in field settings especially by non-clinicians in general population surveys and in cross-cultural settings [35–37]. Perceived mental health was coded as “good” and “poor”.

#### Difficulty in activities of daily living - ADL

Difficulty in activities of daily living (ADL) included having any difficulty with the following six activities: (a) walking across a room, (b) dressing, (c) bathing, (d) eating, (e) getting in and out of bed, and (f) toileting. Responses for the six items (1 = *yes*; 0 = *no*) were summed (range: 0–6) (alpha value for internal consistency was 0.87). Older individuals who struggled with any activity for more than three months were labeled “having difficulties” and otherwise “no.”

#### Difficulty in instrumental activities of daily living – IADL

Difficulty in instrumental activities of daily living (IADL) was assessed by asking respondents to indicate the difficulty they encounter when performing the following seven activities: grocery shopping, preparing meals, making phone calls, taking medication, doing household chores, managing finances, and getting oneself to an otherwise unfamiliar location [34] (alpha value for internal consistency was 0.88). Each item response was coded as 0 for “no difficulty” or 1 for “any difficulty.” Those who reported trouble with any of these activities for more than three months were labeled “having difficulty.” Otherwise, they were categorized as having “no difficulty.” ADL and IADL are considered as measures of functional health and a prolonged difficulty in any of the items refer to individuals’ dependence on others and/or instrumental devices [38, 39].

#### Physical inactivity

Physical activity included vigorous activities such as running or jogging, swimming, going to a health center or gym, cycling, or digging with a spade or shovel, heavy lifting, chopping, farm work, fast bicycling, and cycling with loads; and moderate activities such as playing outdoor games, sports, exercises, jogging and doing yoga. Those who reported none of the respective activities at least once in a week (responses were every day, more than once a week, once a week, one to three times in a month and hardly ever or never) were categorised into yes representing physical inactivity and otherwise no [40]. These measurements of physical activity were adopted from other aging surveys such as the US Health and Retirement Study (HRS) and the China Health, Aging, and Retirement Longitudinal Study (CHARLS) to enable cross-country comparisons and the list of activities was modified to the Indian context to include those that are common in the country.

### Main explanatory variable

The main explanatory variable was self-reported age at onset of chronic illnesses among older adults. The variable was categorized into ‘early-onset’ and ‘late-onset’ of each chronic disease, ‘early onset of single morbidity’, ‘early onset of multimorbidity’, ‘late onset of single morbidity, and ‘late onset of multimorbidity’. The age at onset of each chronic diseases was categorized into ‘early-onset’ with a cut-off of 50 years or early ( = < 50 years of age), and ‘late-onset’ if diagnosed after the age of 50 years (> 50 years of age). In the study, respondents were asked, “Has any health professional ever diagnosed you with the following diseases?”. The diseases were: (1) Hypertension; (2) Diabetes; (3) Heart attack or myocardial infarction; (4) Chronic heart diseases; (5) Stroke; (6) Cancer; (7) Chronic lung diseases; (8) Arthritis or rheumatism; (9) Osteoporosis or other bone/joint diseases; and (10) Psychiatric disease. Respondents were also asked following questions for specific disease, “When you were first diagnosed with particular disease?” The responses, from which the age at onset of each chronic disease was estimated, were in years (in exact year when the disease was diagnosed), before a particular number of years (year at respondents’ birth plus number of years), and in respondent’s age at the time of diagnosis (current year during survey subtracted by age at diagnosis).

### Other covariates

Age was coded as 60–69 years, 70–79 years, and 80 + years. Sex was coded as male and female. Education was recoded as no formal education, primary, secondary, and higher. Marital status was coded as currently in a union, and currently not a union. Living arrangements were coded as living alone, living with spouse and other living arrangements. Working status was coded as never worked, currently working, currently not working, and retired. Alcohol use, smoking tobacco, and chewing tobacco were coded as ‘no’ and ‘yes’. The BMI was computed by dividing the weight (in kilograms) by the square of the height (in meters). BMI was coded according to the criteria of the World Health Organisation’s classification; as underweight (< 18.5 kg/m2), normal weight (18.5–24.9 kg/m2), overweight (25.0–29.9 kg/m2), and obesity (≥ 30.0 kg/m2). The monthly per capita expenditure quintile or consumption quintile was categorized into five quintiles, poorest, poor, middle, rich, and richest. Religion was categorized as Hindu, Muslim, Christian, and Others. The social group (caste) was categorized as Scheduled Castes (SC), Scheduled Tribes (ST), Other Backward Classes (OBC), and others. The ‘other’ category in caste is identified as having high social status. The place of residence was coded as urban, and rural. The regions were categorized as North, Central, East, Northeast, West, and South.

### Analysis

Bivariate analysis (cross-tabulation) and Chi-square test was conducted to examine the prevalence of early and late onset of chronic conditions and their associations with the selected variables. Additionally, multivariable binary logistic regression analysis [41] was used to establish the association between the outcomes (poor perceived physical/mental health, functional difficulty and physical inactivity) and explanatory variables.

The binary logistic regression model is usually put into a more compact form as follows:

$$\text{L}\text{o}\text{g}\text{i}\text{t} \left[\text{P}\left(\text{Y}=1\right)\right]={\beta }_{0}+\beta *X$$

The parameter ${\beta }_{0}$ estimates the log odds of outcome variables for the reference group, while $\beta$ estimates the maximum likelihood, the differential log odds of outcome variables associated with a set of predictors X, as compared to the reference group. The survey weights were applied during the analysis to account for sample clustering and present population estimates. All the analyses were conducted using Stata version 15.1 [42].

## Results

Table 1 presents the socio-demographic characteristics of the study participants. A proportion of 10.77% of the participants were in the age group of 80 years or older in this study. About 52.02% of the sample population were females. A large proportion of the sample of female, male and overall participants (69.72%, 36.29%, and 53.68% respectively) had no education. Moreover, majority of male, female and overall sample population belonged to rural areas (66.7%, 65.06%, and 65.87% respectively).

**Table 1:** Sample characteristics (n = 31,386)

Variables	Male	Female	Total
Age			
60–69 years	8961 (59.35)	10,013 (61.18)	18,974 (60.3)
70–79 years	4545 (30.1)	4556 (27.84)	9101 (28.93)
80 + years	1592 (10.54)	1797 (10.98)	3389 (10.77)
Sex			
Male			15,098 (47.98)
Female			16,366 (52.02)
Education			
No formal education	5479 (36.29)	11,410 (69.72)	16,889 (53.68)
Primary	3361 (22.26)	2479 (15.15)	5840 (18.56)
Secondary	4249 (28.14)	1857 (11.35)	6106 (19.41)
Higher	2009 (13.31)	620 (3.79)	2629 (8.36)
Marital status			
Currently in a union	12,398 (82.12)	7522 (45.96)	19,920 (63.31)
Currently not in a union	2700 (17.88)	8844 (54.04)	11,544 (36.69)
Living arrangements			
Living alone	365 (2.42)	1257 (7.68)	1622 (5.16)
Living with spouse	3739 (24.76)	2476 (15.13)	6215 (19.75)
Other living arrangements	10,994 (72.82)	12,633 (77.19)	23,627 (75.09)
Work status			
Never worked	759 (5.03)	8025 (49.03)	8784 (27.92)
Currently working	5979 (39.6)	5011 (30.62)	10,990 (34.93)
Currently not working	6044 (40.03)	2953 (18.04)	8997 (28.59)
Retired	2316 (15.34)	377 (2.3)	2693 (8.56)
Consumption quintile			
Poorest	3035 (20.1)	3449 (21.07)	6484 (20.61)
Poor	3068 (20.32)	3409 (20.83)	6477 (20.59)
Middle	3064 (20.29)	3352 (20.48)	6416 (20.39)
Rich	2990 (19.8)	3180 (19.43)	6170 (19.61)
Richest	2941 (19.48)	2976 (18.18)	5917 (18.81)
Religion			
Hindu	11,078 (73.37)	11,959 (73.07)	23,037 (73.22)
Muslim	1804 (11.95)	1927 (11.77)	3731 (11.86)
Christian	1468 (9.72)	1682 (10.28)	3150 (10.01)
Others	748 (4.95)	798 (4.88)	1546 (4.91)
Social group			
SC	2448 (16.21)	2692 (16.45)	5140 (16.34)
ST	2436 (16.13)	2737 (16.72)	5173 (16.44)
OBC	5781 (38.29)	6105 (37.3)	11,886 (37.78)
Others	4433 (29.36)	4832 (29.52)	9265 (29.45)
Residence			
Urban	5021 (33.26)	5718 (34.94)	10,739 (34.13)
Rural	10,077 (66.74)	10,648 (65.06)	20,725 (65.87)
Region			
North	2799 (18.54)	3013 (18.41)	5812 (18.47)
Central	2155 (14.27)	2107 (12.87)	4262 (13.55)
East	2863 (18.96)	2894 (17.68)	5757 (18.3)
Northeast	1782 (11.8)	1970 (12.04)	3752 (11.92)
South	3546 (23.49)	4032 (24.64)	7578 (24.08)
West	1953 (12.94)	2350 (14.36)	4303 (13.68)

SC: Scheduled caste; ST: Scheduled tribe; OBC: Other backward classes

Table 2 and Table 3 present the percentage distribution of older adults by early and late onset of chronic conditions by background characteristics. A total of 4.54% of the sample population had early onset of hypertension, whereas 27.99% had late onset of hypertension. On the other hand, 2.29% of the participants had early onset of diabetes, whereas 11.91% had late onset of diabetes. The prevalence of early onset of lung disease and arthritis was 1.46% and 1.64% respectively; whereas the prevalence of late onset of lung disease and arthritis was 6.95% and 14.28% respectively. A total of 7.29% of the participants had late onset of CVD (heart attack, heart disease, and stroke).

**Table 2:** Early onset of chronic diseases, frequency and percentage prevalence (%), (n = 31,386)

Age	Hypertension	Diabetes	Heart attack	Heart disease	Stroke	Cancer	Lung disease	Arthritis	Osteoporosis	Psychiatric diseases
60–69 years	1012 (5.49)	475 (2.58)	63 (0.34)	54 (0.3)	62 (0.33)	22 (0.12)	320 (1.74)	391 (2.12)	98 (0.53)	80 (0.44)
70–79 years	372 (3.95)	231 (2.46)	5 (0.06)	8 (0.09)	6 (0.06)	32 (0.34)	104 (1.11)	98 (1.04)	15 (0.16)	19 (0.2)
80 + years	42 (1.17)	13 (0.36)	2 (0.05)	0 (0.01)	8 (0.23)	0 (0)	33 (0.92)	25 (0.7)	5 (0.15)	5 (0.15)
Sex										
Male	428 (2.89)	325 (2.19)	39 (0.27)	26 (0.17)	42 (0.29)	9 (0.06)	236 (1.6)	160 (1.08)	49 (0.33)	57 (0.39)
Female	997 (6.01)	395 (2.38)	31 (0.18)	37 (0.23)	33 (0.2)	44 (0.27)	221 (1.33)	354 (2.14)	70 (0.42)	47 (0.28)
Education										
No formal education	421 (2.36)	165 (0.93)	16 (0.09)	32 (0.18)	38 (0.22)	12 (0.07)	201 (1.13)	259 (1.46)	53 (0.3)	49 (0.27)
Primary	211 (3.82)	96 (1.75)	10 (0.19)	8 (0.14)	14 (0.25)	32 (0.58)	123 (2.23)	96 (1.73)	34 (0.62)	20 (0.37)
Secondary	604 (10.73)	296 (5.26)	27 (0.48)	16 (0.28)	15 (0.27)	6 (0.1)	80 (1.41)	111 (1.97)	28 (0.5)	26 (0.46)
Higher	189 (7.78)	162 (6.66)	17 (0.68)	7 (0.3)	8 (0.33)	3 (0.14)	54 (2.2)	49 (2)	4 (0.15)	9 (0.37)
Marital status										
Currently in a union	820 (4.25)	450 (2.33)	61 (0.32)	52 (0.27)	50 (0.26)	23 (0.12)	316 (1.64)	338 (1.75)	101 (0.52)	66 (0.34)
Currently not in a union	604 (5)	269 (2.23)	9 (0.07)	11 (0.09)	25 (0.21)	30 (0.25)	141 (1.17)	176 (1.46)	18 (0.15)	39 (0.32)
Living arrangements										
Living alone	60 (3.35)	21 (1.2)	2 (0.1)	1 (0.06)	6 (0.33)	0 (0)	19 (1.06)	17 (0.97)	0 (0.02)	5 (0.29)
Living with spouse	324 (5.18)	157 (2.52)	22 (0.35)	21 (0.34)	17 (0.27)	6 (0.09)	83 (1.33)	124 (1.99)	31 (0.5)	22 (0.35)
Other living arrangements	1041 (4.46)	541 (2.32)	46 (0.2)	41 (0.17)	53 (0.23)	48 (0.2)	355 (1.52)	373 (1.6)	87 (0.37)	77 (0.33)
Work status										
Never worked	701 (8.42)	301 (3.61)	13 (0.16)	23 (0.28)	13 (0.16)	35 (0.42)	114 (1.37)	174 (2.09)	48 (0.57)	21 (0.26)
Currently working	356 (3.13)	180 (1.59)	24 (0.22)	17 (0.15)	38 (0.33)	10 (0.09)	185 (1.63)	168 (1.48)	29 (0.26)	37 (0.32)
Currently not working	230 (2.44)	139 (1.47)	22 (0.23)	19 (0.2)	21 (0.22)	8 (0.09)	111 (1.18)	132 (1.4)	35 (0.37)	41 (0.44)
Retired	138 (6.04)	100 (4.36)	11 (0.46)	4 (0.17)	4 (0.17)	0 (0.01)	47 (2.06)	41 (1.78)	7 (0.32)	5 (0.21)
Alcohol use										
No	1382 (4.68)	699 (2.37)	65 (0.22)	61 (0.21)	74 (0.25)	53 (0.18)	436 (1.48)	490 (1.66)	115 (0.39)	103 (0.35)
Yes	35 (2.11)	16 (0.98)	5 (0.28)	2 (0.13)	2 (0.12)	0 (0)	17 (1.03)	17 (1.03)	3 (0.19)	2 (0.09)
Smoke tobacco										
No	1320 (5.01)	670 (2.54)	63 (0.24)	58 (0.22)	65 (0.25)	51 (0.19)	387 (1.47)	440 (1.67)	97 (0.37)	83 (0.32)
Yes	96 (1.97)	45 (0.93)	7 (0.14)	5 (0.1)	11 (0.22)	2 (0.04)	67 (1.37)	67 (1.38)	21 (0.44)	21 (0.44)
Chew tobacco										
No	1221 (5.19)	627 (2.67)	60 (0.25)	45 (0.19)	54 (0.23)	48 (0.2)	338 (1.44)	383 (1.63)	93 (0.4)	71 (0.3)
Yes	194 (2.52)	88 (1.15)	10 (0.13)	19 (0.24)	22 (0.29)	5 (0.07)	115 (1.5)	125 (1.62)	25 (0.33)	34 (0.44)
Body mass index										
Underweight	422 (2.97)	253 (1.78)	35 (0.25)	22 (0.15)	31 (0.22)	39 (0.27)	202 (1.42)	204 (1.44)	68 (0.48)	48 (0.34)
Normal	114 (1.52)	20 (0.27)	2 (0.02)	13 (0.17)	17 (0.23)	1 (0.02)	98 (1.31)	55 (0.73)	19 (0.26)	29 (0.38)
Overweight	458 (9.95)	192 (4.16)	14 (0.29)	11 (0.24)	14 (0.31)	11 (0.24)	77 (1.67)	122 (2.66)	9 (0.2)	6 (0.14)
Obese	283 (18.42)	190 (12.37)	11 (0.74)	5 (0.32)	3 (0.2)	0 (0)	24 (1.59)	54 (3.52)	12 (0.77)	4 (0.25)
Consumption quintile										
Poorest	165 (2.41)	54 (0.79)	10 (0.14)	11 (0.16)	24 (0.35)	1 (0.02)	63 (0.92)	90 (1.31)	16 (0.24)	21 (0.31)
Poor	192 (2.82)	106 (1.55)	13 (0.2)	7 (0.11)	9 (0.14)	5 (0.08)	97 (1.41)	90 (1.32)	30 (0.44)	16 (0.23)
Middle	233 (3.6)	124 (1.92)	18 (0.28)	16 (0.25)	18 (0.28)	9 (0.14)	102 (1.58)	106 (1.64)	21 (0.32)	28 (0.43)
Rich	379 (6.26)	148 (2.45)	17 (0.28)	10 (0.17)	6 (0.1)	27 (0.45)	105 (1.74)	111 (1.83)	27 (0.45)	26 (0.44)
Richest	455 (8.78)	287 (5.53)	12 (0.23)	18 (0.35)	19 (0.36)	10 (0.2)	90 (1.74)	117 (2.26)	24 (0.46)	14 (0.26)
Religion										
Hindu	1175 (4.53)	596 (2.3)	57 (0.22)	53 (0.2)	59 (0.23)	50 (0.19)	364 (1.41)	424 (1.63)	100 (0.39)	87 (0.33)
Muslim	135 (3.93)	65 (1.9)	8 (0.23)	7 (0.2)	8 (0.23)	2 (0.06)	48 (1.38)	72 (2.08)	13 (0.37)	10 (0.3)
Christian	56 (6.26)	29 (3.28)	4 (0.45)	2 (0.23)	4 (0.46)	1 (0.08)	27 (3)	10 (1.17)	2 (0.19)	6 (0.66)
Others	59 (5.13)	28 (2.48)	1 (0.09)	1 (0.11)	4 (0.38)	0 (0)	18 (1.6)	9 (0.75)	4 (0.38)	1 (0.12)
Social group										
SC	149 (2.51)	55 (0.92)	10 (0.17)	14 (0.24)	15 (0.25)	4 (0.07)	79 (1.32)	86 (1.44)	11 (0.19)	17 (0.29)
ST	34 (1.34)	16 (0.63)	2 (0.08)	1 (0.03)	1 (0.05)	2 (0.07)	29 (1.15)	17 (0.68)	4 (0.14)	5 (0.18)
OBC	765 (5.41)	412 (2.92)	35 (0.25)	20 (0.14)	32 (0.23)	12 (0.09)	207 (1.46)	219 (1.55)	53 (0.38)	44 (0.31)
Others	476 (5.45)	236 (2.7)	23 (0.26)	28 (0.32)	28 (0.32)	35 (0.4)	142 (1.63)	192 (2.2)	51 (0.58)	38 (0.44)
Residence										
Urban	873 (9.55)	509 (5.56)	40 (0.44)	19 (0.21)	21 (0.23)	33 (0.36)	181 (1.98)	173 (1.89)	46 (0.5)	30 (0.33)
Rural	552 (2.48)	211 (0.95)	30 (0.13)	44 (0.2)	55 (0.25)	20 (0.09)	276 (1.24)	342 (1.54)	73 (0.33)	74 (0.33)
Region										
North	149 (3.75)	71 (1.78)	7 (0.18)	7 (0.17)	6 (0.16)	3 (0.08)	58 (1.46)	38 (0.95)	16 (0.41)	18 (0.47)
Central	153 (2.32)	54 (0.81)	7 (0.11)	14 (0.22)	7 (0.11)	2 (0.03)	67 (1.02)	43 (0.65)	13 (0.19)	10 (0.15)
East	168 (2.26)	71 (0.96)	9 (0.11)	28 (0.38)	21 (0.28)	7 (0.1)	106 (1.42)	172 (2.3)	28 (0.37)	28 (0.38)
Northeast	41 (4.33)	8 (0.87)	1 (0.09)	2 (0.2)	1 (0.1)	0 (0.01)	5 (0.58)	5 (0.54)	1 (0.11)	1 (0.14)
South	623 (8.88)	365 (5.2)	20 (0.28)	7 (0.1)	14 (0.2)	30 (0.43)	123 (1.75)	116 (1.65)	43 (0.61)	32 (0.46)
West	291 (5.39)	150 (2.78)	26 (0.49)	4 (0.08)	26 (0.49)	10 (0.19)	97 (1.8)	141 (2.61)	18 (0.33)	14 (0.26)
**Overall**	**1,425 (4.54)**	**720 (2.29)**	**70 (0.22)**	**63 (0.20)**	**76 (0.24)**	**53 (0.17)**	**457 (1.46)**	**515 (1.64)**	**119 (0.38)**	**104 (0.33)**

SC: Scheduled caste; ST: Scheduled tribe; OBC: Other backward classes

**Table 3:** Late onset of chronic diseases, frequency and percentage prevalence (%), (n = 31,386)

Age	Hypertension	Diabetes	Heart attack	Heart disease	Stroke	Cancer	Lung disease	Arthritis	Osteoporosis	Psychiatric diseases
60–69 years	4694 (25.46)	2188 (11.87)	442 (2.4)	299 (1.62)	335 (1.82)	98 (0.53)	939 (5.09)	2374 (12.87)	712 (3.86)	355 (1.92)
70–79 years	2919 (31.06)	1216 (12.94)	452 (4.81)	158 (1.69)	308 (3.28)	42 (0.44)	890 (9.47)	1616 (17.2)	394 (4.2)	247 (2.63)
80 + years	1173 (32.99)	333 (9.37)	111 (3.13)	61 (1.72)	121 (3.41)	28 (0.78)	351 (9.87)	494 (13.9)	246 (6.92)	147 (4.13)
Sex										
Male	3679 (24.83)	1829 (12.34)	571 (3.86)	242 (1.64)	437 (2.95)	80 (0.54)	1085 (7.32)	1755 (11.85)	565 (3.81)	331 (2.23)
Female	5106 (30.81)	1909 (11.52)	434 (2.62)	276 (1.67)	327 (1.98)	87 (0.53)	1095 (6.61)	2728 (16.46)	787 (4.75)	417 (2.52)
Education										
No formal education	4358 (24.46)	1301 (7.3)	304 (1.7)	223 (1.25)	378 (2.12)	77 (0.43)	1239 (6.95)	2486 (13.95)	788 (4.42)	457 (2.57)
Primary	1739 (31.54)	799 (14.49)	208 (3.77)	102 (1.84)	148 (2.68)	32 (0.58)	436 (7.91)	799 (14.49)	246 (4.46)	128 (2.32)
Secondary	1738 (30.9)	1077 (19.15)	351 (6.25)	131 (2.33)	166 (2.95)	31 (0.56)	440 (7.82)	925 (16.44)	225 (4.01)	118 (2.1)
Higher	950 (39.07)	560 (23.02)	142 (5.82)	63 (2.58)	73 (3)	27 (1.11)	66 (2.7)	274 (11.26)	93 (3.82)	45 (1.85)
Marital status										
Currently in a union	5052 (26.19)	2395 (12.42)	645 (3.34)	348 (1.8)	455 (2.36)	99 (0.51)	1222 (6.33)	2482 (12.86)	749 (3.88)	421 (2.18)
Currently not in a union	3734 (30.87)	1342 (11.09)	360 (2.98)	171 (1.41)	310 (2.56)	68 (0.56)	958 (7.92)	2002 (16.55)	604 (4.99)	327 (2.7)
Living arrangements										
Living alone	530 (29.58)	205 (11.42)	27 (1.5)	24 (1.35)	34 (1.88)	2 (0.13)	118 (6.59)	307 (17.12)	110 (6.15)	51 (2.85)
Living with spouse	1615 (25.82)	859 (13.73)	219 (3.5)	99 (1.58)	130 (2.08)	26 (0.41)	393 (6.29)	851 (13.6)	244 (3.9)	144 (2.3)
Other living arrangements	6641 (28.45)	2673 (11.45)	759 (3.25)	395 (1.69)	601 (2.58)	139 (0.6)	1669 (7.15)	3326 (14.25)	998 (4.28)	553 (2.37)
Work status										
Never worked	2870 (34.45)	1216 (14.59)	337 (4.04)	175 (2.1)	162 (1.95)	40 (0.48)	619 (7.43)	1379 (16.55)	395 (4.74)	247 (2.97)
Currently working	3362 (29.59)	1362 (11.99)	380 (3.35)	196 (1.73)	428 (3.76)	78 (0.68)	1009 (8.88)	1850 (16.29)	591 (5.2)	307 (2.7)
Currently not working	1701 (18.08)	658 (6.99)	136 (1.45)	83 (0.89)	87 (0.93)	20 (0.22)	427 (4.54)	970 (10.31)	285 (3.03)	130 (1.38)
Retired	853 (37.28)	501 (21.91)	152 (6.63)	64 (2.79)	87 (3.82)	29 (1.27)	126 (5.51)	283 (12.39)	81 (3.56)	64 (2.8)
Alcohol use										
No	8433 (28.55)	3593 (12.16)	971 (3.29)	498 (1.69)	735 (2.49)	167 (0.56)	2068 (7)	4274 (14.47)	1290 (4.37)	705 (2.39)
Yes	293 (17.57)	118 (7.05)	29 (1.77)	15 (0.9)	25 (1.49)	1 (0.04)	97 (5.83)	181 (10.83)	57 (3.42)	41 (2.48)
Smoke tobacco										
No	7626 (28.94)	3318 (12.59)	800 (3.04)	428 (1.62)	595 (2.26)	137 (0.52)	1697 (6.44)	3863 (14.66)	1184 (4.49)	622 (2.36)
Yes	1099 (22.64)	391 (8.05)	201 (4.14)	86 (1.77)	163 (3.36)	30 (0.62)	467 (9.62)	594 (12.23)	162 (3.35)	126 (2.6)
Chew tobacco										
No	6879 (29.25)	3017 (12.83)	827 (3.52)	404 (1.72)	583 (2.48)	139 (0.59)	1675 (7.12)	3420 (14.54)	1048 (4.45)	551 (2.34)
Yes	1845 (24.02)	691 (9)	173 (2.26)	110 (1.43)	175 (2.27)	29 (0.37)	489 (6.37)	1036 (13.49)	299 (3.89)	197 (2.57)
Body mass index										
Underweight	3889 (27.42)	1558 (10.99)	418 (2.95)	226 (1.59)	327 (2.31)	68 (0.48)	773 (5.45)	1824 (12.86)	503 (3.55)	304 (2.14)
Normal	1232 (16.47)	294 (3.94)	83 (1.12)	82 (1.1)	103 (1.38)	27 (0.36)	695 (9.29)	805 (10.77)	293 (3.92)	180 (2.41)
Overweight	1850 (40.17)	1095 (23.77)	188 (4.08)	107 (2.32)	115 (2.51)	32 (0.71)	204 (4.42)	902 (19.59)	256 (5.56)	107 (2.32)
Obese	734 (47.76)	332 (21.6)	220 (14.33)	38 (2.45)	28 (1.85)	12 (0.75)	220 (14.3)	435 (28.31)	106 (6.9)	24 (1.56)
Consumption quintile										
Poorest	1596 (23.35)	619 (9.06)	138 (2.01)	75 (1.1)	116 (1.7)	15 (0.22)	416 (6.08)	763 (11.16)	260 (3.8)	165 (2.42)
Poor	1761 (25.76)	599 (8.76)	136 (1.99)	91 (1.33)	160 (2.35)	24 (0.35)	469 (6.85)	923 (13.49)	300 (4.39)	148 (2.17)
Middle	1795 (27.71)	690 (10.64)	182 (2.81)	110 (1.7)	162 (2.5)	24 (0.38)	397 (6.13)	888 (13.71)	236 (3.64)	144 (2.23)
Rich	1894 (31.28)	950 (15.69)	178 (2.94)	117 (1.94)	157 (2.6)	41 (0.67)	374 (6.18)	997 (16.47)	296 (4.89)	162 (2.68)
Richest	1739 (33.56)	880 (16.97)	371 (7.16)	124 (2.4)	169 (3.26)	64 (1.23)	525 (10.12)	912 (17.61)	261 (5.03)	128 (2.47)
Religion										
Hindu	6973 (26.91)	3040 (11.73)	820 (3.17)	368 (1.42)	599 (2.31)	127 (0.49)	1777 (6.86)	3717 (14.35)	1068 (4.12)	619 (2.39)
Muslim	1124 (32.72)	388 (11.3)	109 (3.18)	114 (3.33)	99 (2.89)	22 (0.64)	283 (8.24)	552 (16.07)	152 (4.44)	87 (2.54)
Christian	271 (30.18)	157 (17.48)	32 (3.57)	18 (1.99)	26 (2.89)	5 (0.57)	56 (6.27)	110 (12.24)	48 (5.36)	23 (2.61)
Others	419 (36.51)	152 (13.29)	43 (3.77)	18 (1.58)	40 (3.52)	13 (1.17)	64 (5.58)	104 (9.09)	84 (7.29)	19 (1.64)
Social group										
SC	1496 (25.1)	521 (8.75)	142 (2.38)	71 (1.2)	159 (2.67)	26 (0.44)	433 (7.27)	809 (13.58)	305 (5.11)	181 (3.03)
ST	431 (16.88)	140 (5.48)	16 (0.64)	16 (0.64)	43 (1.69)	9 (0.35)	143 (5.58)	245 (9.57)	56 (2.21)	47 (1.85)
OBC	3886 (27.5)	1830 (12.95)	527 (3.73)	231 (1.63)	309 (2.19)	56 (0.4)	1047 (7.41)	2209 (15.63)	603 (4.27)	331 (2.34)
Others	2973 (34)	1246 (14.25)	320 (3.66)	199 (2.28)	254 (2.9)	76 (0.87)	557 (6.37)	1220 (13.95)	388 (4.44)	190 (2.17)
Residence										
Urban	3405 (37.23)	1870 (20.45)	528 (5.77)	217 (2.38)	257 (2.81)	70 (0.76)	605 (6.61)	1481 (16.19)	454 (4.96)	202 (2.21)
Rural	5381 (24.19)	1867 (8.39)	477 (2.14)	301 (1.35)	508 (2.28)	97 (0.44)	1576 (7.08)	3002 (13.5)	899 (4.04)	546 (2.45)
Region										
North	1372 (34.56)	429 (10.81)	111 (2.79)	84 (2.11)	76 (1.93)	29 (0.73)	364 (9.17)	357 (8.99)	245 (6.16)	58 (1.46)
Central	1257 (19)	493 (7.46)	98 (1.48)	42 (0.63)	128 (1.94)	18 (0.27)	440 (6.66)	465 (7.04)	281 (4.25)	119 (1.81)
East	2087 (27.99)	674 (9.04)	230 (3.09)	170 (2.28)	221 (2.97)	41 (0.55)	478 (6.41)	1195 (16.02)	206 (2.77)	227 (3.05)
Northeast	303 (32.34)	75 (8)	5 (0.51)	26 (2.81)	24 (2.59)	5 (0.49)	40 (4.25)	35 (3.72)	8 (0.88)	18 (1.88)
South	2146 (30.58)	1305 (18.6)	304 (4.34)	137 (1.95)	145 (2.06)	27 (0.38)	544 (7.75)	1453 (20.72)	373 (5.31)	255 (3.64)
West	1622 (30.04)	762 (14.11)	257 (4.76)	60 (1.11)	170 (3.15)	48 (0.89)	314 (5.82)	978 (18.11)	240 (4.44)	71 (1.31)
**Overall**	**8786 (27.99)**	**3737 (11.91)**	**1005 (3.20)**	**518 (1.65)**	**765 (2.44)**	**167 (0.53)**	**2181 (6.95)**	**4483 (14.28)**	**1353 (4.31)**	**748 (2.38)**

SC: Scheduled caste; ST: Scheduled tribe; OBC: Other backward classes

**Fig. 2:**
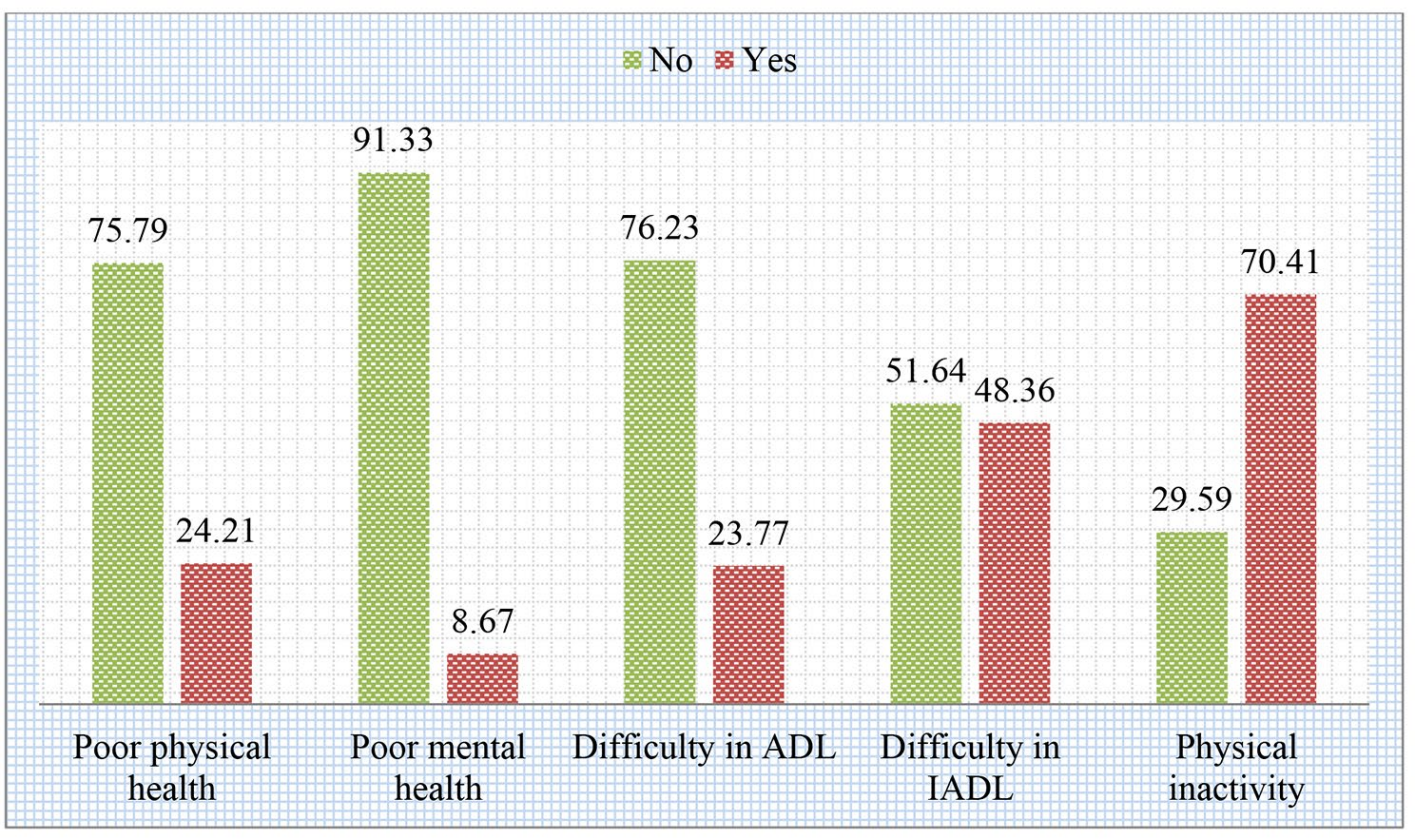
Prevalence of poor self-perceived physical and mental health, functional limitations and physical inactivity

**Fig. 3:**
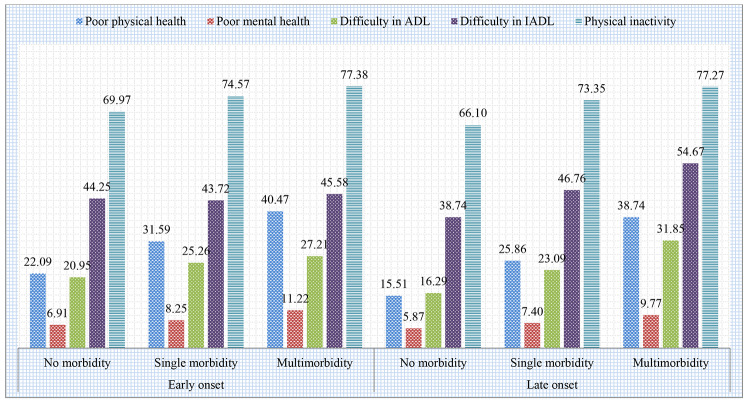
Prevalence of adverse health outcomes by early and late onset of single and multimorbidity status among older adults

Figure 2 depicts the prevalence of health outcomes in this study. Overall, 24.21% of the sample population had poor self-perceived physical health, whereas 8.67% of participants had poor self-perceived mental health. The prevalence of difficulty in ADL, difficulty in IADL, and physical inactivity was 23.77%, 48.36%, and 70.41% respectively. Further, Fig. 3 presents the prevalence of adverse health outcomes by early/late onset of single and multimorbidity status among older adults. Older adults with early and late onset of multimorbidity had higher prevalence of all the adverse health outcomes in this study.

Table 4 presents the multivariable logistic regression estimates of physical, mental and functional health outcomes by early and late onset of all morbidity in this study. Respondents with late onset of all chronic diseases were significantly associated with poor perceived physical health; while those with early onset of hypertension (adjusted odds ratio [AOR]: 1.60, confidence interval [CI]: 1.11–2.32), heart attack (AOR: 2.65, CI: 1.22–5.78), lung disease (AOR: 3.18, CI: 2.33–4.34), psychiatric diseases (AOR: 3.44, CI: 1.72–6.88), and arthritis (AOR: 2.58, CI: 1.92–3.46) were more likely to have poor perceived physical health than those without any chronic disease. Individuals with late onset of cancer were more likely to have poor perceived physical health (AOR: 2.62, CI: 1.58–4.36) than those without chronic disease.

**Table 4:** Association of early and late onset of specific diseases with health outcomes

Onset of morbidity	Poor perceived physical health	Poor perceived mental health	Functional difficulty (ADL)	Functional difficulty (IADL)	Physical inactivity
	AOR (95% CI)	AOR (95% CI)	AOR (95% CI)	AOR (95% CI)	AOR (95% CI)
**Early onset of each disease (Ref: no disease)**					
Hypertension	1.60* (1.11–2.32)	1.79** (1.23–2.61)	1.06 (0.74–1.53)	1.74** (1.12–2.71)	1.23 (0.85–1.79)
Diabetes	1.47 (0.83–2.59)	1.61 (0.93–2.80)	0.69 (0.37–1.28)	1.95* (1.16–3.29)	1.22 (0.79–1.89)
Heart attack	2.65** (1.22–5.78)	2.15 (0.82–5.62)	2.23* (1.01–4.90)	1.01 (0.52–1.96)	1.06 (0.56–2.02)
Heart disease	1.34 (0.57–3.15)	0.69 (0.20–2.35)	3.65** (1.29–10.3)	4.30*** (1.88–9.87)	0.85 (0.29–2.45)
Stroke	2.07 (0.82–5.21)	3.39*** (1.52–7.56)	3.76*** (1.48–9.54)	3.48*** (1.47–8.19)	1.23 (0.50–3.02)
Cancer	0.67 (0.11–4.02)	0.56 (0.10–3.04)	1.39 (0.28–6.82)	2.77 (0.83–9.26)	1.66 (0.47–5.82)
Lung disease	3.18*** (2.33–4.34)	1.45 (0.77–2.74)	1.38 (0.91–2.08)	1.05 (0.75–1.47)	1.32 (0.90–1.94)
Arthritis	2.58*** (1.92–3.46)	2.12*** (1.42–3.18)	2.38*** (1.71–3.31)	1.98*** (1.43–2.74)	1.31 (0.89–1.93)
Osteoporosis	2.01 (0.96–4.22)	1.98 (0.39–10.1)	3.92*** (1.65–9.32)	2.09* (1.09–4.02)	1.34 (0.92–1.98)
Psychiatric diseases	3.44*** (1.72–6.88)	1.81 (0.76–4.31)	1.40 (0.61–3.26)	2.00 (0.93–4.30)	0.59 (0.28–1.22)
**Late onset of each disease (Ref: no disease)**					
Hypertension	1.82*** (1.63–2.03)	1.69*** (1.45–1.97)	1.33** (1.18–1.51)	1.25** (1.13–1.39)	1.08 (0.95–1.23)
Diabetes	2.00*** (1.71–2.36)	1.27* (1.01–1.59)	1.04 (0.87–1.23)	1.27* (1.06–1.54)	0.96 (0.78–1.18)
Heart attack	1.59* (1.01–2.53)	1.66* (1.09–2.52)	1.38 (0.89–2.14)	1.68* (1.13–2.50)	1.35 (0.97–1.87)
Heart disease	2.61*** (1.99–3.43)	1.48* (1.01–2.18)	1.35 (0.98–1.84)	1.54** (1.16–2.06)	0.91 (0.65–1.24)
Stroke	3.60*** (2.77–4.67)	2.32*** (1.61–3.35)	2.22*** (1.72–2.87)	2.97*** (2.20–4.01)	1.58** (1.09–2.30)
Cancer	2.62*** (1.58–4.36)	1.65 (0.82–3.31)	1.39 (0.81–2.40)	0.94 (0.55–1.61)	2.04** (1.02–4.11)
Lung disease	2.03*** (1.61–2.56)	1.27 (0.98–1.64)	1.01 (0.80–1.27)	1.69*** (1.35–2.10)	1.23* (1.01–1.49)
Arthritis	1.58*** (1.34–1.86)	1.34* (1.11–1.62)	1.92*** (1.64–2.25)	1.95*** (1.64–2.32)	1.35 (0.97–1.87)
Osteoporosis	2.29*** (1.87–2.80)	2.06*** (1.55–2.74)	1.61*** (1.29–2.01)	1.57*** (1.28–1.94)	1.17 (0.99–1.37)
Psychiatric diseases	3.51*** (2.73–4.52)	2.89*** (2.11–3.94)	3.41*** (2.68–4.34)	2.95*** (2.29–3.81)	0.90 (0.71–1.13)

Notes: *p < 0.05, **p < 0.01, ***p < 0.001; AOR: Adjusted odds ratio, adjusted for all the selected covariates; CI: Confidence interval; ADL: Activities of daily living; IADL: Instrumental activities of daily living

The odds of poor perceived mental health were higher for the respondents with early as well as late onset of hypertension, stroke, and arthritis. Individuals with early onset of hypertension (AOR: 1.79, CI: 1.23–2.61), stroke (AOR: 3.39, CI: 1.52–7.56), and arthritis (AOR: 2.12, CI: 1.42–3.18) had 1.7, 3.3 and 2.1 times higher odds of poor perceived mental health, respectively than those without any morbidity. The result also presents that individuals with late onset of hypertension (AOR: 1.69, CI: 1.45–1.97), stroke (AOR: 2.32, CI: 1.61–3.35), arthritis (AOR: 1.34, CI: 1.11–1.62), diabetes (AOR: 1.27, CI: 1.01–1.59), heart attack (AOR: 1.66, CI: 1.09–2.52) and heart disease (AOR: 1.48, CI: 1.01–2.18) had higher odds of poor perceived mental health as compared to those without chronic disease.

Individuals with early onset of heart attack (AOR: 2.23, CI: 1.01–4.90) and arthritis (AOR: 2.38, CI: 1.71–3.31) had more than two times higher odds of reporting ADL difficulty, while those with early onset of heart disease (AOR: 3.65, CI: 1.29–10.30), stroke (AOR: 3.76, CI: 1.48–9.54), and osteoporosis (AOR: 3.92, CI: 1.65–9.32) were having more than three times higher odds of reporting ADL difficulty than those with no morbidity. Similarly, the result indicated that participants with late onset of hypertension (AOR: 1.33, CI: 1.18–1.51) and stroke (AOR: 2.22, CI: 1.72–2.87) had higher odds of suffering from ADL difficulty than the respondents without any morbidity. Individuals with late-onset of arthritis, osteoporosis, and psychiatric diseases had higher odds of ADL than those without any morbidity.

Individuals with early as well as late onset of diabetes, hypertension, arthritis, and osteoporosis were more likely to have difficulty in IADL. Participants with early onset of hypertension (AOR: 1.74, CI: 1.12–2.71), diabetes (AOR: 1.95, CI: 1.16–3.29), and arthritis (AOR: 1.98, CI: 1.43–2.74) had higher odds of IADL difficulty. Additionally, respondents with early onset of heart disease (AOR: 4.30, CI: 1.88–9.87), stroke (AOR: 3.48, CI: 1.47–8.19), and osteoporosis (AOR: 2.09, CI: 1.09–4.02) were having more than 4.3, 3.4, and 2 times higher odds of IADL difficulty, respectively, than those with no morbidity. Moreover, individuals with late onset of hypertension, diabetes, arthritis, osteoporosis, lung disease, and heart disease were more likely to have difficulty in IADL, while those with late onset of stroke (AOR: 2.97, CI: 2.20–4.01) and psychiatric disease (AOR: 2.95, CI: 2.29–3.81) had more than two times higher odds of IADL disability compared to those without any chronic condition.

Respondents with late onset of stroke (AOR: 1.58, CI: 1.09–2.30) and chronic lung disease (AOR: 1.23, CI: 1.01–1.49) were more likely to be physically inactive than the respondents without any morbidity. Further, individuals with late onset of cancer had more than two times (AOR: 2.04, CI: 1.02–4.11) higher odds of being physically inactive than those with no morbidity.

Table 5 presents the multivariable logistic regression estimates of physical, mental and functional health outcomes by early and late onset of single and multimorbidity. The odds of poor perceived physical health, poor perceived mental health, and IADL difficulty increased as chronic conditions increased from single to multimorbidity in early as well as late onset. Individuals with early onset of single morbidity were more likely to report poor perceived physical health (AOR: 1.72, CI: 1.30–2.27)), poor perceived mental health (AOR: 1.41, CI: 1.07–1.85), ADL difficulty (AOR: 1.33, CI: 1.06–1.67) and IADL disability (AOR: 1.40, CI: 1.08–1.82); while those with early onset of multimorbidity had more than two times higher odds of poor perceived physical health (AOR: 2.17, CI: 1.01–4.69), poor perceived mental health (AOR: 2.31, CI: 1.32–4.06) and IADL disability (AOR: 2.57, CI: 1.63–4.05) than individuals without chronic condition.

**Table 5:** Association of early and late onset of morbidity with health outcomes

Onset of morbidity	Poor perceived physical health	Poor perceived mental health	Functional difficulty (ADL)	Functional difficulty (IADL)	Physical inactivity
	AOR (95% CI)	AOR (95% CI)	AOR (95% CI)	AOR (95% CI)	AOR (95% CI)
**Early onset of morbidity**					
No morbidity	Ref.	Ref.	Ref.	Ref.	Ref.
Single morbidity	1.72*** (1.30–2.27)	1.41* (1.07–1.85)	1.33* (1.06–1.67)	1.40* (1.08–1.82)	1.10 (0.85–1.42)
Multimorbidity	2.17* (1.01–4.69)	2.31** (1.32–4.06)	1.31 (0.64–2.67)	2.57*** (1.63–4.05)	1.31 (0.88–1.97)
**Late onset of morbidity**					
No morbidity	Ref.	Ref.	Ref.	Ref.	Ref.
Single morbidity	1.80*** (1.61–2.02)	1.36*** (1.15–1.60)	1.34*** (1.17–1.53)	1.38*** (1.24–1.54)	1.08 (0.96–1.23)
Multi-morbidity	3.44*** (2.97–3.98)	2.21*** (1.83–2.67)	1.91*** (1.63–2.24)	2.08*** (1.81–2.40)	1.17* (1.01–1.37)
Observation	27,748	27,754	27,758	27,719	27,748
Pseudo R2	0.09	0.06	0.08	0.11	0.20

Notes: *p < 0.05, **p < 0.01, ***p < 0.001; AOR: Adjusted odds ratio, adjusted for all the selected covariates; CI: Confidence interval; ADL: Activities of daily living; IADL: Instrumental activities of daily living

Similarly, participants with late onset of single morbidity had higher odds of poor perceived physical health, poor perceived mental health, ADL and IADL difficulty, whereas individuals with late onset of multimorbidity were more likely to report poor perceived physical health (AOR: 3.44, CI: 2.97–3.98), poor perceived mental health (AOR: 2.21, CI: 1.83–2.67), physical inactivity (AOR: 1.17, CI: 1.01–1.37), ADL (AOR: 1.91, CI: 1.63–2.24), and IADL difficulty (AOR: 2.08, CI: 1.81–2.40) than those without chronic disease. Individuals with early as well as late-onset of multimorbidity had more than two times higher odds of reporting poor physical health, poor mental health and difficulty in IADL compared with individuals without chronic disease.

## Discussion

Given the paucity of research on interactions between age and chronic diseases with major health outcomes, the current study aimed to examine the association of early and late onset of chronic single and multiple morbidities with self-perceived physical and mental health, functional limitations and physical inactivity among older Indian adults. The study found that the odds of poor perceived physical health, poor perceived mental health, and functional difficulty increased as chronic conditions increased from single to multimorbidity in case of both early as well as late onset of chronic conditions. Also, respondents with late onset of stroke and arthritis had higher odds of all the selected adverse physical, mental and functional health outcomes.

Multimorbidity and single chronic conditions are correlated with subjective health expressed as self-perceived health [9, 10]. Our research also suggested a significant relationship between self-perceived health and the early and late onset of various chronic diseases such as hypertension, heart disease, stroke, cancer, lung disease and arthritis. A study conducted by Griffith et al. (2019) reported that multimorbidity was associated with disability and self-rated physical health [43]. This finding may partially be explained by the reduced quality of life due to the long term care needs associated with chronic conditions among individuals [44]. Given the differential impact of chronic diseases on self-perceived health across various socio-demographic groups [45–47], complex needs of multimorbid older patients should be prioritized in policies. Prior study has also suggested that self-perceived health, which is responsive to chronic diseases or multimorbidity, can be considered a proxy for health biomarkers and a barometer of physiologic conditions [48].

Furthermore, recent studies among older Indian also revealed that the number of chronic conditions are positively associated with functional difficulty [49, 50] and higher prevalence of depression [51]. Concordantly, the present study found that individuals with early as well as late onset of multimorbidity had higher odds of poor perceived metal health and difficulty in IADL compared to those without any chronic conditions. Another previous study focusing on the age of onset of chronic conditions found that individuals with early onset multimorbidity or both early and late multimorbidity were more likely to have physical disability, poor perceived general and mental health and physical frailty [52]. Similarly, our study revealed that individuals with early onset of single morbidity were more likely to report ADL difficulty. These findings corroborate the evidence of a scoping review on the consequences of chronic diseases associated with old age [53]. Being unable to perform simple tasks might cause elderly individuals to become isolated and lose confidence [54]. Numerous studies have demonstrated that being able to perform a variety of tasks, such as obtaining food and cooking, gives older persons a pleasant sense of independence [55].

The finding presents that the late onset of chronic conditions is associated with poor perceived mental health among older adults is in concordance with findings from previous studies. The psychological dimensions of chronic conditions are frequently ignored when medical care is considered. Patients with chronic diseases frequently need to adjust their ambitions, way of life, and professions. Many people refuse to acknowledge their situation before coping with it. However, some people have prolonged distress and may develop psychiatric illnesses, most frequently anxiety or depression [56]. Our study has further revealed that the odds of poor perceived mental health were higher for the respondents with early as well as late onset of hypertension, arthritis and stroke and late onset of diabetes, in line with previous research findings. A study has underlined that due to fluctuations in blood sugar, whether high or low, a person may experience several significant symptoms, including mood swings [57]. According to another study, after a stroke, the brain’s neuroplasticity has been found to either speed up recovery or cause unanticipated behavioural changes that result in mental diseases [58]. Depression, anxiety, exhaustion, sleep difficulties, and emotionalism are among the mental diseases that are frequently linked to stroke [59]. Moreover, our study also explored that late onset of psychiatric disease and both early and late onset of hypertension, diabetes, arthritis, heart diseases, and stroke had higher odds of IADL. In line with the present study, previous studies have demonstrated that hypertension, diabetes, arthritis, stroke, and psychiatric diseases have positive association with IADL [29, 60–62].

Physical activity is an established but notably under-utilized health promotion strategy. Unsurprisingly, verifying the previous findings, the present study observed that individuals with chronic diseases are at a greater risk of physical inactivity, which may contribute to some of the harmful effects of chronic conditions [63]. Evidence has highlighted that physical activity partially mediates the effect of chronic diseases on a number of health outcomes essential to the quality of life [33]. Besides, regular physical activity improves muscle strength and postural balance [64], reduces the risk of depression [65] and cognitive impairment [66], which directly help older persons maintain their mobility and also has indirect benefits, including lowering the risk of fractures and falls [67–69]. Similarly, physical activity can play an important role to reduce the risk of multimorbidity among those who are overweight/obese and those who have an at-risk waist circumference or at-risk waist-hip ratio [70]. Therefore, we recommend paying closer attention to physical activity as a potential health promotion strategy for older persons with chronic diseases.

### Policy implications and direction for future research

The findings of this study revealed that individuals with early and late onset of chronic diseases, and multimorbidity had higher likelihoods of experiencing adverse health outcomes such as poor perceived physical and mental health, functional limitations and physical inactivity. This emphasizes the importance of public health support to young adults diagnosed with chronic diseases and multiple chronic conditions to prevent adverse health consequences as they grow older. Healthcare professionals can benefit from gaining a deeper understanding of the specific combinations of early or late onset of chronic diseases and multimorbidity that have varying levels of impact on physical, mental and functional outcomes among older adults.

Patients diagnosed with early-onset of hypertension, diabetes, lung disease, cardiovascular diseases, arthritis, and psychiatric diseases face distinct challenges throughout the disease trajectory. Healthcare professionals should emphasize on prevention, early detection of chronic disease and early interventions to reduce disease burden and improve health outcomes. By recognizing and addressing these conditions and age at the onset, clinicians can provide more comprehensive and effective care for older adults. Promoting awareness among individuals about possible adverse health outcomes of early and late onset chronic diseases and multmorbidity, encouraging them to seek medical assessment, and actively participating in screening programs are essential for improving early detection of chronic disease. Moreover, additional longitudinal research is necessary to understand the impact of early and late onset chronic diseases and multimorbidity on the health outcomes.

### Limitations and strength of the study

Our study has certain limitations to be mentioned. Firstly, due to the data’s cross-sectional nature, we cannot verify the causality in the observed associations. Secondly, classifications of chronic illnesses lack independence. For example, people who have had a stroke are also more prone to developing cardiovascular diseases. Thirdly, self-report of chronic diseases may subject to reporting bias. Lastly, our analyses did not consider several other chronic diseases that could affect older person’s quality of life, such as epilepsy and migraine headaches. The large nationally representative sample of older adults is one of the study’s strengths. Also, it is the first study that has specifically examined the association of early and late onset of chronic single and multiple morbidities with major health outcomes.

## Conclusions

The present study revealed that early and/or late onset of chronic single and/or multiple morbidities significantly predicted poor self-perceived physical and mental health, functional limitations and physical inactivity among older Indian adults. Findings imply that identifying people with early and late-onset chronic conditions might help target the subpopulations more vulnerable to negative mental, physical and functional health outcomes. The findings further suggest that late onset of chronic diseases such as cancer and stroke and multi-morbidity had significant associations with physical inactivity that may help identify high risk groups for screening and support. Further studies are needed to determine the types of interventions most beneficial for older adults with specific chronic conditions for improving mental, physical and functional health outcomes.

## Data Availability

The data are available at The Gateway to Global Aging Data (https://g2aging.org/ ).
